# The Anesthesiology Clerkship: A Requisite Experience in Medical Education

**DOI:** 10.31486/toj.20.0094

**Published:** 2020

**Authors:** Eric I. Ly, Blas S. Catalani, Steven D. Boggs, Kathleen E. McGreevey, Amelia B. Updegraff, Joy L. Steadman

**Affiliations:** ^1^ Department of Anesthesiology, The University of Tennessee Health Science Center, Memphis, TN; ^2^ Department of Anesthesiology, The University of Oklahoma Health Sciences Center, Oklahoma City, OK; ^3^ Department of Anesthesiology, The University of Utah School of Medicine, Salt Lake City, UT

## INTRODUCTION

Modern medical education has a serious deficit—a curriculum gap that exists between essential anesthesiology education and the associated clinical exposure. Meanwhile, the shortage of anesthesiologists in the US healthcare workforce continues to grow.^1-4^ While important (yet focused) data from the Association of American Medical Colleges (AAMC) and the National Resident Matching Program (NRMP) help shed light on this curriculum gap,^5-9^ large-scale investigations of US-based medical school anesthesiology curriculum requirements are lacking in the published literature. Our aim is to highlight the importance of such a curriculum requirement and encourage relevant decision makers to investigate the merits of a mandatory rotation in anesthesiology in US-based medical education institutions.

In our view, an anesthesiology rotation ought to be a required experience for every medical student. If an anesthesiology rotation were a mandatory experience, future physicians would become more aware of the fact that anesthesiologists are resources of extensive expertise that can be engaged for patient care optimization. Another potential benefit is the development of procedural skills fundamental to the practice of anesthesia yet broadly applicable to other disciplines as well (eg, airway management) that can improve the clinical performance of postgraduate residents. Greene et al surveyed 500 graduating medical students and residents at Harvard Medical School from 2008 to 2009, and 30% responded that they felt uncomfortable performing invasive procedures such as managing airways and achieving vascular access.^10^ Further, students can reinforce and refine effective interdisciplinary communication skills learned elsewhere in their medical school curricula, as such communication is essential to the practice of anesthesiology.

Anesthesiology rotations also offer students unique insight into the perioperative management of surgical patients and not just the elements exclusive to surgical management of specific medical conditions. Such a rotation can also provide broad exposure to the multidisciplinary approach necessary to successfully care for such patients. At this time, however, clinical anesthesiology rotations overwhelmingly remain an elective choice for US medical students. Consequently, a key specialty that routinely interacts with nearly every other specialty in medicine is not a required experience for graduating doctors.

## AN ABSENCE OF AWARENESS

Medical school is the opportune time to develop interest in anesthesiology, as virtually all graduating medical students entering residency training developed their specialty preferences during medical school. Many of these students, however, appear to lack adequate understanding of the scope and proficiencies required of anesthesiologists. A survey of medical students at the Loyola Stritch School of Medicine found that some medical students did not ascribe an integral role in the operating room to anesthesiologists.^11^ This misguided assumption is not limited to US medical students, as international reports by Adudu et al from both Nigeria and Canada have also demonstrated medical students’ unawareness of the considerable overlap between anesthesiology and other medical specialties, with students ultimately stating that an anesthesiology rotation would benefit their learning.^12,13^

Not surprisingly, a structured 4-week educational clerkship improved US-based medical students’ understanding of anesthesiology and increased students’ interest in that specialty.^14^ Galway reported that 96% of the students surveyed stated that the anesthesia clerkship increased their desire to pursue a career in the field.^14^ Watts et al reported that positive role models, especially anesthesiologists, influence career choice.^15^ Murray and Wiisanen reported that anesthesiology exposure during medical school generated greater interest in the field, with 70% of participants in an anesthesiology elective becoming more interested in the field as a career choice.^11^

Anesthesiologists take a multidisciplinary approach to healthcare management, collaborating with clinicians in other specialties to develop treatment plans for patients. This approach has been particularly evident during the 2019 novel coronavirus (COVID-19) pandemic crisis, with anesthesiologists on the frontlines managing critically ill patients.^16,17^ Anesthesiology commonly interfaces with intensive care unit medicine, emergency medicine, and procedural medicine, and many anesthesiologists have specifically redeployed as critical care physicians during the pandemic.^18^ Multidisciplinary skills integration is also a hallmark of the practicing anesthesiologist, and exposure to integrated, team-driven perioperative and critical care resulting in the development of comprehensive care plans is an experience that prepares medical students for the practice of modern medicine, regardless of specialty choice.

## THE EVIDENCE

An exhaustive search of the available literature failed to yield any studies that critically evaluate publicly obtainable data compiled by large medical education organizations to investigate the beneficial aspects of a required clinical rotation in anesthesiology; only smaller, site-specific assessments have been performed.^19-23^ Consequently, we used data provided by the AAMC and the NRMP to examine the level of exposure to anesthesiology in medical school and medical students’ interest in the field.

**Figure 1.**
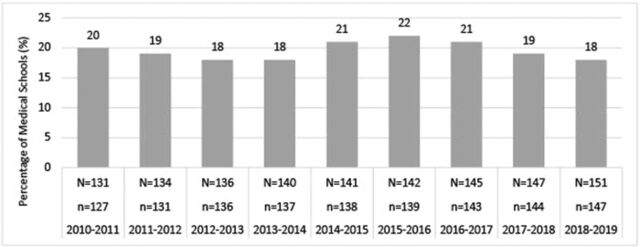
**Percentage of medical schools with separate required anesthesiology clerkship.** Source: Liaison Committee on Medical Education Annual Medical School Questionnaire Part II, 2010-2011 through 2018-2019.^5^ N**,** total number of medical schools that participated in the survey for the given academic year; n, total number of medical schools that responded to the survey item for the given academic year.

**Figure 2.**
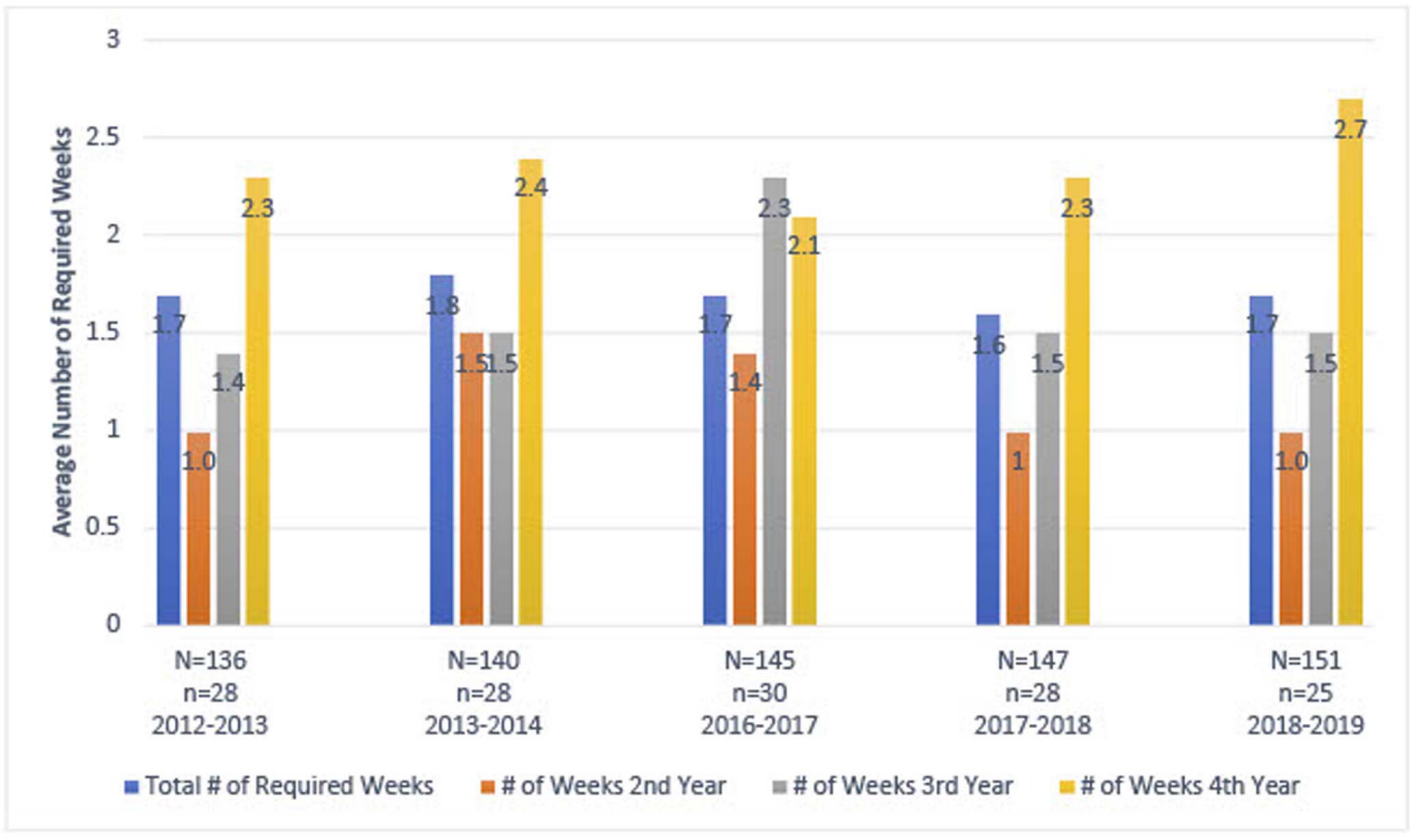
**Average number of required weeks by academic year.** Source: Liaison Committee on Medical Education Annual Medical School Questionnaire Part II, 2012-2013, 2013-2014, 2016-2017, 2017-2018, and 2018-2019 (missing academic years were not reported by the Association of American Medical Colleges).^6^ N**,** total number of medical schools that participated in the survey for the given academic year; n, total number of medical schools that responded to the survey item by providing a number of weeks for any of the categories.

**Figure 3.**
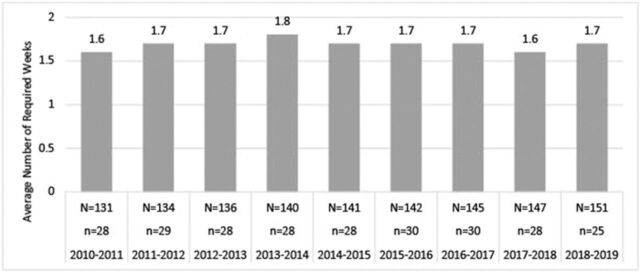
**Average number of required weeks during medical school.** Source: Liaison Committee on Medical Education Annual Medical School Questionnaire Part II, 2010-2011 through 2018-2019.^7^ N**,** total number of medical schools that participated in the survey for the given academic year; n, total number of medical schools requiring one or more total weeks in the given academic year.

AAMC data suggest that medical schools offer inadequate exposure to the field, as approximately 80% of medical schools do not require an independent anesthesiology rotation in a given academic year (Figure 1).^5^ The remainder (approximately 20%) of surveyed schools indicated that students spent on average no more than 2.7 weeks per year in an anesthesiology rotation, with no more than 1.8 weeks on average during their entire medical school career (Figures 2 and 3).^6,7^ Despite such a small contingent of schools requiring a rotation in clinical anesthesiology, data show that medical students’ desire for greater exposure to the field does exist.^11,24^ The Stanford University School of Medicine determined that after an expansion of the elective anesthesiology rotation and revision in curriculum to follow guidelines published by the Society for Education in Anesthesia, demand for the rotation increased and participation nearly tripled after 5 years despite the rotation's elective status.^24^

With regard to continuity of interest in a given specialty, more than 25% of students reported to the AAMC a “same specialty preference” at the start and end of medical school. Of note, among all specialties, anesthesiology had one of the lowest rates of “same specialty preference” at 9.3%, indicating that students have an initial interest in anesthesiology but often pursue a different specialty during their medical education.^9^ Such consequences may be precipitated by the aforementioned limited exposure to the field.^11,20,23,24^

NRMP data reveal a surprising applicant deficit for the anesthesiology specialty. In 2020, of the 2,119 rank list applications submitted for the 1,884 available anesthesiology residency positions, only 1,736 came from US fourth-year medical students (MDs and DOs) (Table),^8^ representing a US-based medical student anesthesiology applicant deficit of 148. The fact that the number of US-trained applicants failed to apply for the total training positions available is concerning, as an applicant surplus (235) only occurs once international medical graduate applicants are included (383). In fact, when considering only US-based medical graduates, anesthesiology as a specialty has experienced a trend in US-based applicant deficits (2016, 2018, and 2020) despite anesthesiology being the sixth largest specialty by total residency positions available nationwide. Therefore, increasing the national prevalence of anesthesiology rotations is an essential approach to ensuring adequate exposure to the discipline and resolving the applicant deficit, especially considering that such a rotation is already an interest among students who cite that the clerkship served a valuable purpose^20,23,24^ and improved their attitudes toward anesthesiologists.^11,25^

**Table.** Anesthesiology Residency Positions Offered and Applicants Who Ranked Anesthesiology First^8^

		Applicants Who Ranked Anesthesiology First (Difference)	
		
Year	Positions Offered	International Medical Graduates and US-Based Medical School Applicants	US-Based Medical School Applicants Only (MD and DO)
2016	1,696	1,771 (+75)	1,309 (–387)
2017	1,743	1,826 (+83)	NR
2018	1,840	2,004 (+164)	1,425 (–415)
2019	1,862	2,188 (+326)	NR
2020	1,884	2,119 (+235)	1,736 (–148)

Note: Exclusive data for US-based medical school applicants was not reported by the National Resident Matching Program in 2017 and 2019; consequently, the reported applicant surplus in those 2 years does not reflect US-based medical applicants only as the pooled data include international medical graduates.

NR, not reported.

## DISCUSSION

The authors are of the opinion that an emphasis on the importance of anesthesiology demands a bottom-up approach beginning with the breadth of educational exposure in medical school. Early focus on student perceptions is important because students largely have an underappreciation for anesthesiology's scope of practice and believe that anesthesia providers merely administer drugs to ensure that a patient is unconscious during surgery.^25^

Several studies have investigated the benefit of a required rotation to produce informed students who are aware of the scope and value of an anesthesiologist.^19-23^ Patel showed that pairing anesthesiology with a general surgery rotation, even for 1 week, significantly improved the perioperative medicine education and knowledge of students. Students even ascribed added value to the general surgery rotation itself.^21^ The modern approach to medicine—in which patient care is team-based—necessitates knowledge of every specialty's role and scope,^19-21^ particularly for such a large field as anesthesiology that supplies the sixth most residency positions in the NRMP. At the University of Florida College of Medicine, Euliano et al determined that more than 50% of each group (students, anesthesiology faculty, and faculty from each major specialty) considered the topics taught in the required anesthesiology curriculum important for all future physicians.^19^ In a survey of third-year medical students at Beth Israel Deaconess Medical Center in Boston, MA, nearly 80% reported that the required 1-week anesthesiology rotation helped them gain important skills for whatever specialty they chose. Such studies reinforce the notion that a substantive anesthesiology experience early in medical school benefits the education of students entering all medical specialties, albeit on a site-specific basis.^19-21^ Consequently, anesthesiology rotations should become required elements in the curriculum on a national scale.

Our assessment of the current climate of medical education utilized publicly available databases to reveal a widespread insufficiency of anesthesiology education in the medical education experience. To benefit all future clinicians and improve the quality of their patient care (regardless of specialty), we feel it is necessary to improve the breadth of education for all US-based medical students. We are of the opinion that increasing exposure to anesthesiology during medical school gives all medical students the opportunity to gain greater understanding of an anesthesiologist's value in the perioperative setting and will lead to an improvement in multispecialty collaboration and communication between physicians, a reduction in perioperative medical errors, and ultimately an improvement in patient outcomes.
